# Gut microbiota and fecal 2-methylbutyric acid in coronary heart disease: a cross-sectional study

**DOI:** 10.1038/s41598-026-49930-0

**Published:** 2026-04-22

**Authors:** Wilanee Dechkhajorn, Supachai Topanurak, Kriengchai Prasongsukarn, Surachet Benjathummarak, Chantira Sutthikornchai, Yaowapa Maneerat

**Affiliations:** 1 Department of Tropical Pathology, Faculty of Tropical Medicine, Mahidol University, 420/6 Ratchawithee Road, Bangkok, 10400 Thailand; 2 Department of Molecular Tropical Medicine and Genetics, Faculty of Tropical Medicine, Mahidol University, Bangkok, 10400 Thailand; 3 Phramongkutklao Hospital and College of Medicine, Bangkok, 10400 Thailand; 4 Center of Excellence for Antibody Research (CEAR), Faculty of Tropical Medicine, Mahidol University, Bangkok, 10400 Thailand; 5 Department of Protozoology, Faculty of Tropical Medicine, Mahidol University, Bangkok, 10400 Thailand

**Keywords:** Coronary heart disease, Gut microbiota, Branched short-chain fatty acid, 2-methylbutyric acid, *Eubacterium*, Biomarkers, Cardiology, Diseases, Medical research, Microbiology

## Abstract

The influence of gut microbiota diversity and/or metabolite levels on the development of coronary heart disease (CHD) is unclear. This cross-sectional study investigated whether specific gut microbiota and/or their metabolites are associated with CHD development. The study cohort comprised 55 Thai male volunteers, including 24 normal controls (N), 17 patients with hyperlipidemia (H), and 14 with CHD. We used gas chromatography-mass spectrometry to measure the short-chain fatty acid (SCFA) and branched short-chain fatty acid (BSCFA) contents of fecal water extracts. We also determined the gut microbiota composition for each group using *16S rRNA* gene sequencing. Although comprising only a minor proportion, an unidentified *Eubacterium* spp. with 100% sequence similarity to *E*. *liposum* was more dominant in CHD than the N (false discovery rate (FDR) = 0.0002) or H groups (FDR = 0.0018). Furthermore, the CHD group exhibited significantly higher levels of 2-methylbutyric acid, a BSCFA, than the N (FDR = 0.0006). In addition, Spearman’s correlation analysis showed a moderately significant association between 2-methylbutyric acid and CHD development (*r* = 0.5037, *p* = 0.0001). In conclusion, our observations revealed that patients with CHD had a prominent abundance of *Eubacterium* and 2-methylbutyric acid. Further in-depth studies are needed to draw definitive conclusions.

**Supplementary Information:**

The online version contains supplementary material available at 10.1038/s41598-026-49930-0.

## Introduction

Gut microbes in humans and animals produce metabolites, particularly short-chain fatty acids (SCFAs), such as acetic, propionic, butyric, and hexanoic acids. They are the fermentation products of indigestible carbohydrates and are involved in regulating normal physiological, metabolic, and immunological functions^1,2^. Additionally, various diseases are linked to the abundance of distinct bacterial species^3^. Thus, dysbiosis, i.e., alterations in gut microbiota composition and function, disrupts the balance between pathogenic and beneficial microbes, as well as their products. Changes in the proportion of SCFAs contribute to metabolic dysfunction, as well as several inflammatory and noncommunicable diseases, including hypertension^4,5^, obesity^6^, hyperlipidemia, atherosclerosis, and cardiovascular disease (CVD)^7–10^. The most common CVD is coronary heart disease (CHD), a complication that arises due to progressive hyperlipidemia and coronary atherosclerosis^11,12^.

During a reduction in the intake of fermentable carbohydrates, increased protein fermentation in the colon enhances the production of branched short-chain fatty acids (BSCFAs), such as isobutyric, isovaleric, 2-methylbutyric, 3-methylvaleric, and 4-methylvaleric acids. These in turn are derived from the proteolytic fermentation of branched-chain amino acids (BCAAs) in the colon by gut microbiota. BCAAs include leucine, isoleucine, and valine, three essential amino acids, which are synthesized by bacteria, fungi, and plants^13^. BSCFAs in the colon of humans and animals adversely affect health, e.g., a dysregulation of adipocyte lipid and glucose metabolism^14^ as well as an enhanced risk of type 2 diabetes mellitus^15,16^ and other metabolic syndromes^17^. Dysbiosis, particularly decreased SCFAs and increased BSCFAs within the gut and circulation, is correlated with the incidence of CVDs^16,18^. However, the impacts of SCFAs and BSCFAs on mechanisms related to such disorders are limited. We hypothesized that CHD, a complication of atherosclerosis, is characterized by altered microbiota signatures—potential biomarkers of CHD. We tested our assumption with a cross-sectional study comparing the diversity and composition of gut microbiota and corresponding SCFA/BSCFA profiles among three groups of male volunteers representing each stage of CHD development: normal controls (N), hyperlipidemia (H), and CHD^19^. We also established an association between specific microbiota and SCFA/BSCFA profiles with CHD development.

## Results and discussion

### Characteristics of normal volunteers and patients

**Fig. 1:**
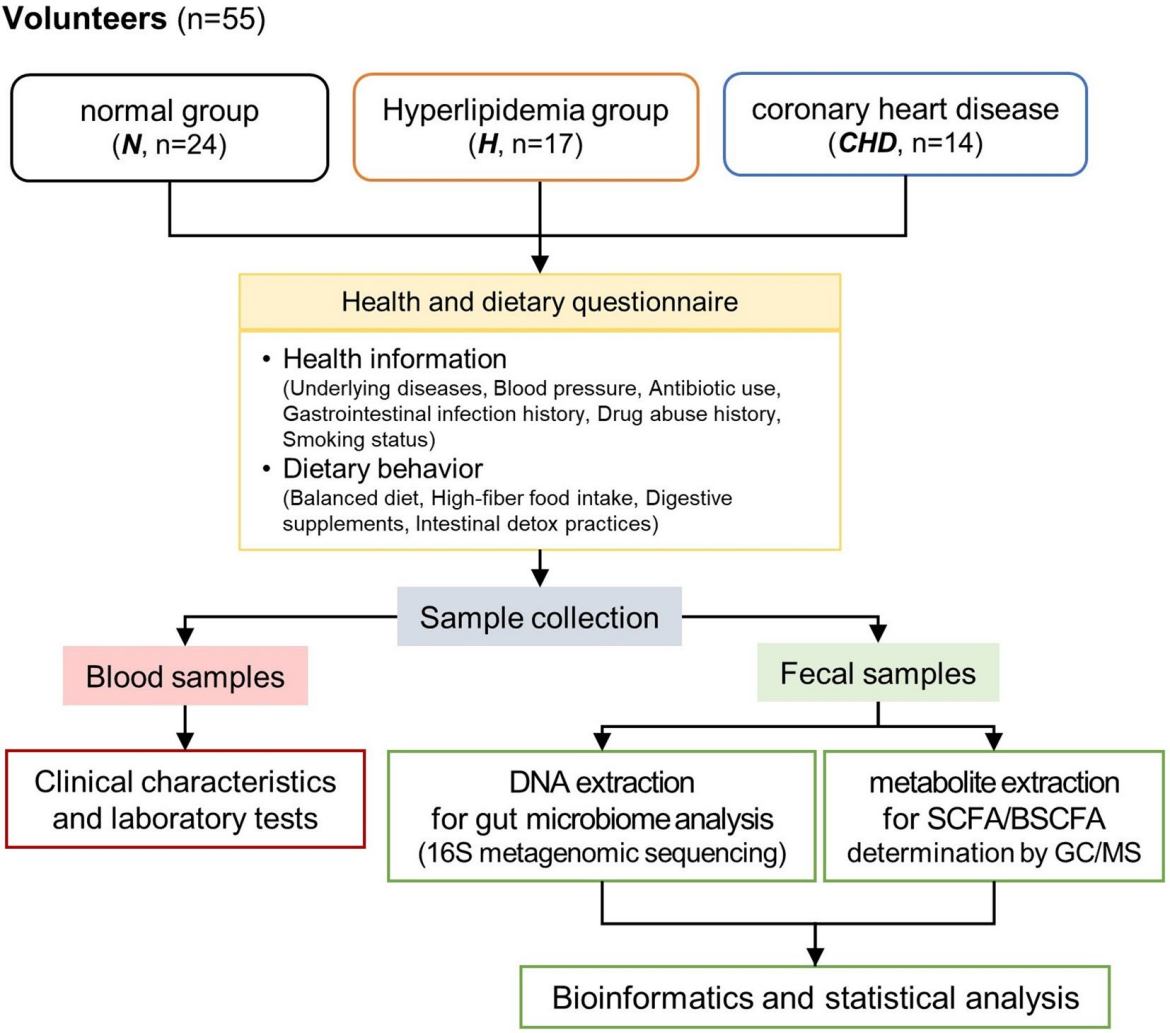
Experimental design and study population.

This cross-sectional study included 24 normal volunteers (N), 17 patients with hyperlipidemia (H), and 14 patients with CHD (Fig. 1). The three groups are representative of the long-term progression of atherosclerosis, from initiation to the development of CHD. Table 1 summarizes the characteristics and clinical manifestations of the participants. The total cholesterol (TC) and low-density lipoprotein (LDL) levels were markedly higher in the H than in the N group. However, the lipid profile of the CHD group fell within normal ranges because these patients were started on lipid and cholesterol-lowering and antiplatelet medication immediately after diagnosis, as appropriate by KP. The CHD patients received Atorvastatin (9 of 14), or Rosuvastatin (5 of 14), with Ezetrol (5 of 14), and aspirin (14 of 14). Previous studies reported that several drugs used for cardiovascular therapy interact with the gut microbiome. Effects of statin in patients with acute coronary syndrome or chronic statin therapy showed partial restoration of gut microbiota homeostasis, including increased abundance of *Bifidobacterium longum*,* Anaerostipes hadrus*, and *Ruminococcus obeum*, along with reduced *Parabacteroides merdae*^20,21^. In addition, She J, et al. 2024, reviewed that statin usage correlates to the increase in abundance of the phylum Verrucomicrobia, and the genera Bacteroides, including *Butyricimonas*, *Mucispirillum*,* Oscillospira*,* Akkermansia*,* Lactobacillus*,* Bifidobacterium*,* Anaerostipes*,* Ruminococcus*,* Eubacterium*, and *Faecalibacterium*, but a decrease in the abundance of the genera *Desulfovibrio* and *Parabacteroides*^22^. The antiplatelet drug, aspirin, used in the CHD patient group, has recently been reported to modulate the gut microbiome. However, the role of aspirin in CVD therapy, which interacts with the gut microbiome, is still obscured^23^.

**Table 1:** General description and clinical manifestations of the participants in each group.

Variables	*N* (*n* = 24)	H (*n* = 17)	CHD (*n* = 14)	*p*-value* (group)	*p*-value**		
					*N* vs. H	*N* vs. CHD	H vs. CHD
Age (year)	48 ± 2.5	45 ± 3.3	69 ± 2.1	< 0.0001	ns	< 0.0001	< 0.0001
Lipid profile							
–TC	178.8 ± 6.3	236.8 ± 6.6	139.5 ± 8.9	< 0.0001	< 0.0001	0.0007	< 0.0001
–TG	134.9 ± 11.5	249.0 ± 44.5	139.5 ± 19.2	0.0058	0.0065	ns	0.0444
–HDL-C^1^	50.5 (32–80)	45 (33–84)	46.5 (38–81)	ns^2^	–	–	–
–LDL	99.3 ± 5.3	143.0 ± 3.9	58.1 ± 7.2	< 0.0001	< 0.0001	< 0.0001	< 0.0001

All patients and controls were Thai male. N, normal; H, hyperlipidemia; and CHD, patients diagnosed with coronary heart disease. Data are shown as Mean ± SEM for normality data and ^1^median (minimum-maximum) for nonparametric. The comparison of values was determined by *one-way ANOVA test and Unpair-*t* test. The comparison of nonparametric was determined by ^2^Kruskal–Wallis test. The α level was set at < 0.05 with a 95% confidence interval. (TC, total cholesterol (mg/dL); TG, triglycerides (mg/dL); HDL-C, high density lipoprotein-cholesterol (mg/dL); LDL, low density lipoprotein (mg/dL)).

In the present study, the CHD group was significantly older than the N and H groups. We could not avoid this limitation because the disease typically develops progressively with advancing age^11^. Focusing on the influence of age in our study, we performed age-adjusted multivariable linear regression analyses with age in the H and CHD groups as a covariate and the N group as the reference^24,25^. The analysis proved that the influence of age on abundance of *Eubacterium* and level of 2-methylbutyric acid are not significantly changed (Supplementary file S1).

### Microbiota composition, SCFA and BSCFA levels, and association with CHD pathogenesis

**Fig. 2:**
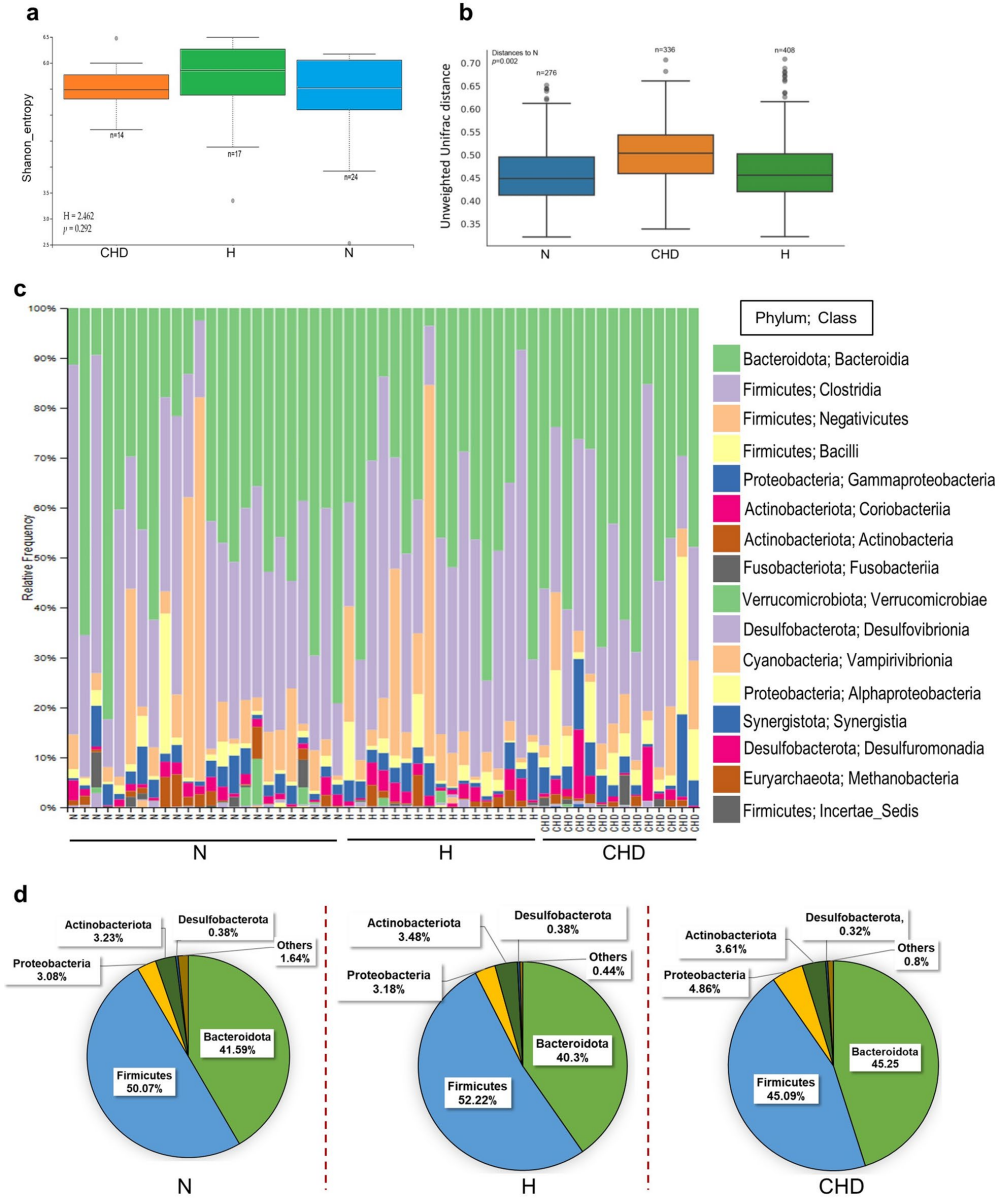
Gut microbiota diversity and taxonomic composition in normal, hyperlipidemia, and CHD groups. Difference in gut microbial diversity and community in normal control (N), hyperlipidemia (H), and coronary heart disease patients (CHD) groups. (**a**) Shannon entropy graph quantifies the biodensity of gut microbes in N, H, and CHD, which are not significantly different. (**b**) Unweighted Unifrac distance graph compares microbe types in N, H, and CHD groups. Types of microbes in CHD groups is significantly more diverse than those in N and H groups. (**c**) Relative abundance of gut microbiota at phylum and class levels in N, H, and CHD groups. Each bar represents anindividual sample, showing similar overall profi les by the top two phyla, Bacteroidota and Firmicutes with inter-individual variation. (**d**) Pie charts showing the average relative abundance of bacterial phyla in N, H, and CHD groups. Firmicutes andBacteroidota are the dominant phyla in all groups.

**Fig. 3:**
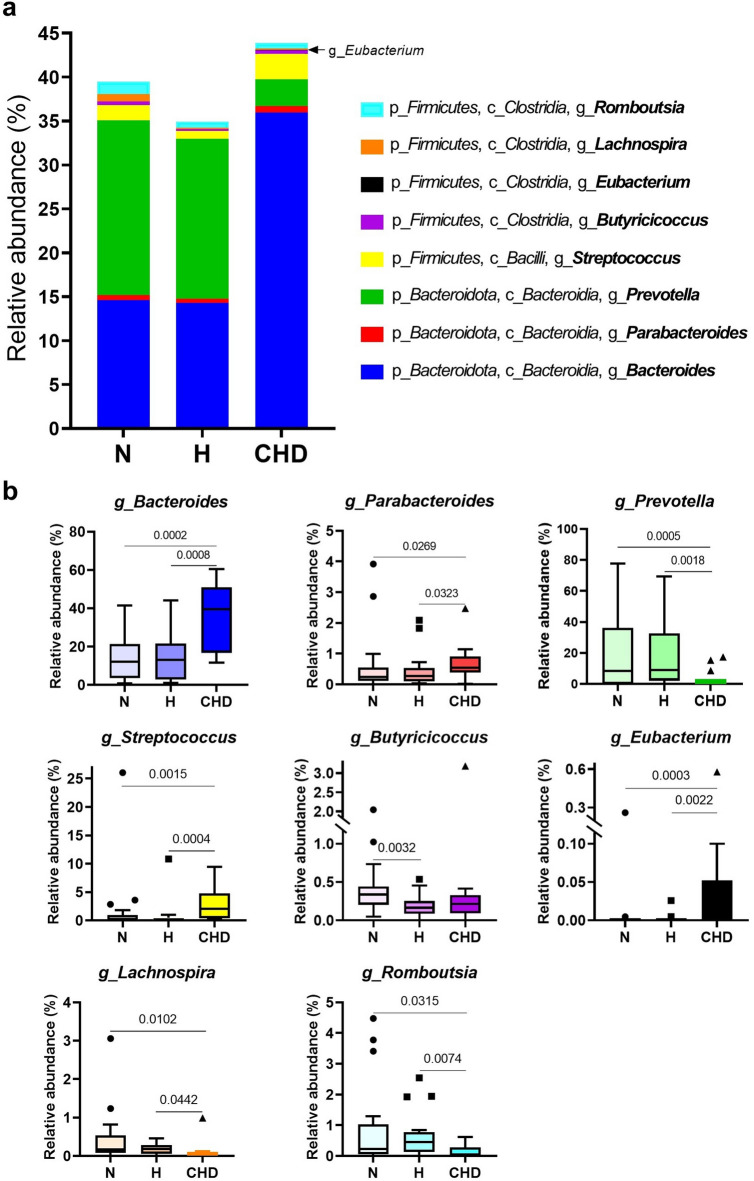
Differential abundance of gut bacterial genera among the N, H, and CHD groups. Significant abundance of genera of gut microbiota in normal control (N), hyperlipidemia (H), and coronary heart disease patients (CHD) groups. (**a**) in bar ratio of percentage proportion; (**b**) comparison of relative abundance among groups. The comparison between groups was determined by an unpaired *t*-test. The α level was set at < 0.05 with a 95% confidence interval.

We compared the composition and abundance of gut microbiota in the N, H, and CHD groups (Fig. 2); their diversity did not differ significantly (Fig. 2a). An unweighted Unifrac distance graph indicating the microbial variety of the three groups (Fig. 2b) revealed a significantly distinct composition of certain microbes in the CHD group compared to the N and H groups (*p* = 0.002). The result was confirmed by a PERMANOVA of the unweighted Unifrac distances (pseudo-F = 2.019, R^2^ = 0.069, *p* = 0.002; 999 permutations), indicating a moderately remarkable variation in microbial community composition among these groups. The gut microbiota consisted of six phyla: Firmicutes, Bacteroidetes, Actinobacteria, Proteobacteria, Fusobacteria, and Verrucomicrobia, with the first two being dominant^26^. The main phyla and classes among the N, H, and CHD groups identified in our study, included Bacteriodota (class: Bacteroidia), Firmicutes (class: Clostridia, Negativicutes, and Bacilli), Proteobacteria (class: Gammaproteobacteria), and Actinobacteriota (class: Coriobacteriia and Actinobacteria) (Fig. 2c). Although the composition among all groups was similar, the abundance and proportion of each was divergent (Fig. 2d). The abundance of *Bacteroides* was higher in the CHD group (45.25%) than that in the N (41.59%) and H (40.3%) groups; in contrast, Firmicutes were less abundant in the former (45.09%) than in the latter (52.22% and 50.07%, respectively). We compared the proportions of the primary gut microbiota genera among the three groups (Fig. 3a; Table 2, and Supplementary file S2), as well as the diversity and abundance of all species within each genus among the three groups. We also analyzed the data to identify any correlations between microbiota and SCFA/BSCFA levels (Supplementary file S3). We observed that the most abundant pathogens among the gut microbiota were similar between the CHD groups and those reported in patients with CVDs and other metabolic diseases^8,17,27^.

**Table 2:** Bacterial genera produce important SCFA and BSCFA.

Phylum/Genus	*N* (*n* = 24)	H (*n* = 17)	CHD (*n* = 14)	*p*	FDR (q)	Sig. pairs	Correlation** with SCFA/BSCFA						
							AA	PA^***^	BA	VA	IBA	2-MBA	IVA***
p_Bacteroidota													
*Bacteroides*	4710 (287–16076)	5025 (435–17076)	15,358 (4496–23456)	0.0007*	**< 0.0001**	N vs. CHD H vs. CHD	− 0.04471 (0.7482)	**− 0.2989** **(0.0281)**			− 0.04982 (0.7205)		− 0.0051 (0.9708)
*Parabacteroides*	94 (0–1522)	107 (11–811)	212 (9–958)	**0.0492**	ns	–	**0.3566** **(0.0081)**			**0.2886** **(0.0343)**	**0.356** **(0.0082)**		**0.3261** **(0.0161)**
*Prevotella*	3272 (0–30126)	3502 (0–26892)	0 (0–6728)	**0.0016**	**0.0024**	N vs. CHD H vs. CHD		**0.5375** **(< 0.0001)**					
p_Firmicutes													
*Streptococcus*	103.5 (3–10111)	78 (13–4205)	796.5 (100–3674)	**0.0012**	**0.0032** **0.0018**	N vs. CHD H vs. CHD	0.1656 (0.2315)						**0.2967** **(0.0294)**
*Butyricicoccus*	132 (19–796)	64 (0–209)	84 (0–1235)	**0.0116**	**0.0096**	N vs. H			− 0.2103 (0.127)				
*Eubacterium*	0 (0–101)	0 (0–10)	2.5 (0–224)	**0.0002**	**0.0003** **0.0009**	N vs. CHD H vs. CHD	0.1995 (0.1481)		− 0.1336 (0.3353)			**0.3947** **(0.0031)**	**0.3456** **(0.0105)**
*Lachnospira*	66 (0–4102)	71 (0–179)	25.5 (0–382)	**0.0238**	**0.0192**	N vs. CHD		0.136 (0.3269)	**0.3406** **(0.0117)**			**− 0.2896** **(0.0336)**	
*Romboutsia*	85.5 (0–6270)	172 (0–988)	17 (0–2775)	**0.0224**	**0.0228**	N vs. CHD	− 0.0685 (0.6226)						

Data are shown as median (minimum-maximum). The comparison of values was determined by the Kruskal–Wallis test or *one-way ANOVA, as appropriate. Multiple comparisons were adjusted using the Benjamini–Hochberg (BH) false discovery rate (FDR) procedure (Q = 0.05). Correlations with SCFA/BSCFA were assessed using **Spearman’s rank correlation coefficient, except where indicated (***), for which Pearson’s correlation coefficient was applied. The α level was set at < 0.05 with a 95% confidence interval. (N, normal; H, hyperlipidemia; and CHD, patients diagnosed with coronary heart disease; AA, Acetic acid; PA, Propionic acid; VA, Valeric acid; IBA, Isobutyric acid; 2-MBA, 2-Methylbutyric acid, IVA, Isovaleric acid).

Bold indicates statistically signifi cant values (*p*< 0.05 and/or FDR (q) < 0.05).

Furthermore, in agreement with previous studies^7,8^, *Bacteroides* was remarkably more abundant in the CHD than in the N and H groups (Supplementary file S2). *Bacteroides*, *Streptococcus*, and *Parabacteroides* exhibited the same trend; however, the abundances of the genera *Prevotella*, *Lachnospira*, and *Romboutsia* demonstrated the opposite pattern (Fig. 3b; Table 2). *R. ilealis* as well as its key metabolite, 2-oxindole-3-acetic acid, markedly suppressed intestinal lipid absorption, potentially alleviating obesity and related metabolic disorders^28^. Furthermore, *R. lituseburensis* can improve endothelial function and lipid metabolism, thereby protecting against atherosclerosis^29^. However, we noted that the abundance and diversity of these genera are common among several other diseases and metabolic disorders^8,17,27,29–32^, and similarly, among all groups in our study.

The limitation in the CHD sample size was small. To reduce the risk of false-positive results or type 1 error, multiple comparisons across both microbial genera and metabolites were analyzed using the Benjamini–Hochberg false discovery rate (FDR) procedure with q = 0.05^33,34^. The results showed that several bacterial genera, including *Bacteroides*,* Prevotella*,* Streptococcus*, and *Eubacterium*, and the target 2-methylbutyric acid levels significantly differ among the groups (all FDR < 0.05). The FDR (q values) was added in Tables 2 and 3, and Supplementary file S3. However, the sample size is still limited by statistical power. We suggest that further studies with a large sample size are needed to confirm the current findings.

**Table 3:** Fecal concentration (ppm) of SCFA and BSCFA in normal, hyperlipidemia and CHD groups.

SCFA/BSCFA	*N* (*n* = 24)	H (*n* = 17)	CHD (*n* = 14)	p* (group)	p**			FDR (q)		
					*N* vs. H	*N* vs. CHD	H vs. CHD	*N* vs. H	*N* vs. CHD	H vs. CHD
SCFA										
Acetic acid^1^	294.0 ± 28.1	301.3 ± 30.3	267.8 ± 33.5	0.7602	0.8649	0.5625	0.4640	0.8631	0.8266	0.8266
Propionic acid^1^	28.4 ± 3.3	27.7 ± 4.8	16.7 ± 3.9	0.1011	0.9036	**0.0331**	0.0930	0.8996	0.1196	0.1196
Butyric acid^1^	35.9 ± 3.6	37.2 ± 4.4	31.3 ± 3.6	0.6015	0.8238	0.4081	0.3188	0.8137	0.6250	0.6250
Valeric acid^1^	8.8 ± 1.0	13.6 ± 2.3	11.3 ± 1.9	0.1110	**0.0390**	0.2126	0.4539	0.1151	0.3731	0.3731
BSCFA										
Isobutyric acid^1^	4.9 ± 0.6	7.7 ± 1.1	8.0 ± 1.1	**0.0194**	**0.0184**	**0.0105**	0.8452	**0.0340**	**0.0340**	0.8229
Isovaleric acid^1^	4.6 ± 0.6	7.5 ± 1.1	8.4 ± 1.3	**0.0128**	**0.0183**	**0.0051**	0.6247	**0.0416**	**0.0214**	0.5662
2-Methylbutyric acid^2^	25.8 (6.6–1039)	505.1 (6.4–747.2)	577.4 (505.6–898.9)	**0.0009**	0.1197	**< 0.0001**	**0.0383**	0.1199	**0.0006**	0.0594

All patients and controls were Thai male. N, normal; H, hyperlipidemia; and CHD, patients diagnosed with coronary heart disease. Data ^1^mean ± SEM for normal distribution data and comparison of values was determined by one-way ANOVA test and Unpaired t-test. ^2^Data are shown as median (minimum-maximum) and comparison of values was determined by *Kruskal-Wallis test and **Mann-Whitney test. The α level was set at < 0.05 with a 95% confidence interval. Multiple comparisons were adjusted using the Benjamini–Hochberg (BH) false discovery rate (FDR) procedure (Q = 0.05). ns = not significant.

Bold indicates statistically signifi cant values (*p*< 0.05 and/or FDR (q) < 0.05).

**Fig. 4:**
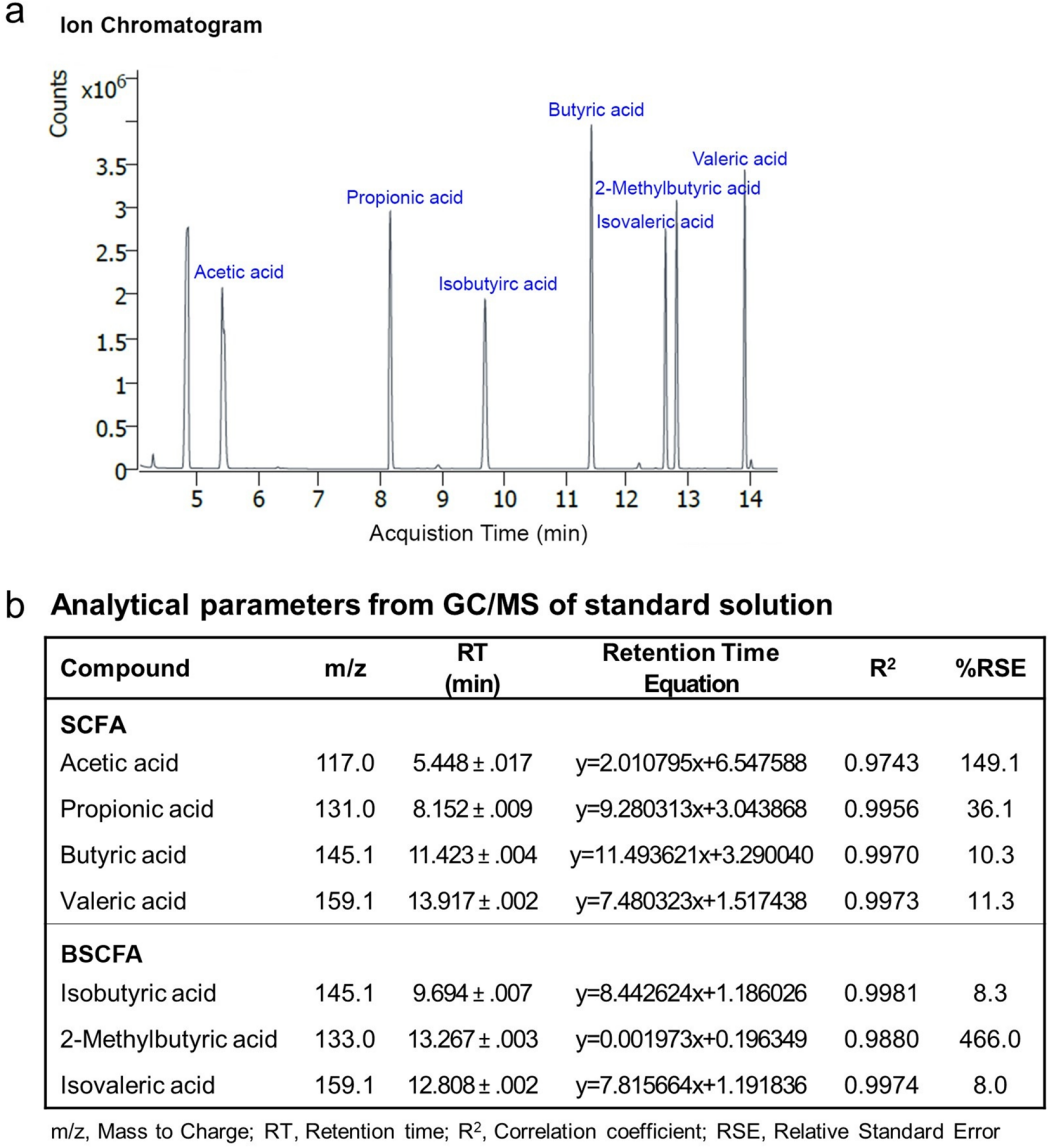
GC/MS identification and analytical parameters of SCFAs and BSCFAs. (**a**) An Ion Chromatogram of 7 SCFA/BSCFA standard mixture shows different peaks in order to acquisition time (minute), correspond to acetic acid, propionic acid, isobutyric acid, butyric acid, isovaleric acid, 2-methylbutyric acid, and valeric acid. (**b**) Analytical parameters from GC/MS of standard solution. m/z, Mass to Charge; RT, Retention time; R^2^, Correlation coefficient; RSE, Relative Standard Error.

**Fig. 5:**
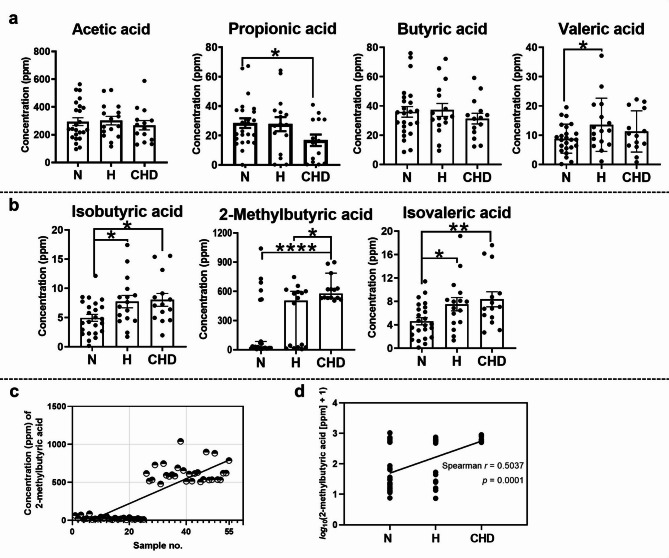
Comparison of fecal SCFA and BSCFAs among N, H, and CHD groups. (**a**) Concentrations of short-chain fatty acids (SCFAs), including acetic acid, propionic acid, butyric acid, and valeric acid. (**b**) Concentrations of branched-chain fatty acids (BSCFAs), including isobutyric acid, 2-methylbutyric acid, and isovaleric acid. Values are presented as mean ± SEM. Statistical comparisons among groups were performed using an unpaired t-test. (**c**) Scatter plot showing the distribution of 2-methylbutyric acid concentrations across samples and its correlation with category groups. (**d**) Correlation analysis was performed using Spearman’s rank correlation coeffi cient. Statistical signifi cance was setat α 0.05 with a 95% CI.

We calculated the concentrations of SCFAs/BSCFAs in fecal samples based on calibration curves plotted employing the signals of the corresponding standards determined by gas chromatography-mass spectrometry (GC-MS) (Fig. 4). Table 3 presents the data for each group as well as the mean ± standard error of the mean (SEM). SCFA levels—acetic, butyric, and valeric acids—did not vary significantly among all groups. However, those of propionic and valeric acid were markedly lower in the CHD and N groups, respectively (Fig. 5a; Table 3). Our comparison of BSCFA contents among the three groups (Fig. 5b) revealed that the levels of 2-methylbutyric acid were significantly greater in the CHD than in the N and H groups. In contrast, the amounts of isovaleric acid, though meager, were remarkably higher in the H and CHD groups than in the N group (Fig. 5b; Table 3). The abundance of eight major genera and their correlations with the corresponding SCFAs/BSCFAs are summarized in Table 2.

Interestingly, the average quantities of fecal 2-methylbutyric acid in the CHD group were increased, approximately ≥ 560 ppm, while those of most others were approximately < 60 ppm (Fig. 5b; Table 3). Thus, the fecal concentration of 2-methylbutyric acid may serve as a discriminant factor of CHD for further elucidation.

Supplementary file S2 summarizes the abundances of gut microbiota genera among all groups. Supplementary file S3 presents the SCFA/BSCFA profiles of crucial genera, along with prominent correlations between specific genera and respective metabolites. The concentration of 2-methylbutyric acid was significantly greater in the CHD than the N and H groups (*p* = 0.0009), and was correlated with *Eubacterium* abundance. Additionally, the level of 2-methylbutyric acid showed a moderate significant association with CHD development (Spearman rho: *r* = 0.5037, *p* = 0.0001).

Notably, *Eubacterium* dominated the CHD but not the N and H groups (Fig. 3b; Table 2). Although it formed only a minor proportion, it was associated with CHD development. The latest iteration of Bergey’s Manual of Systematics of Archaea and Bacteria, as well as NCBI Taxonomy, classified *Eubacterium* to the phylum Firmicutes, order Clostridiales, and family Eubacteriaceae^35^; it is widely distributed across the phylogenetic tree. *Eubacterium* spp. are considered beneficial because they produce abundant amounts of acetic, butyric, and formic acids, but not of propionic acid^36^.

Previous studies reported that a majority of beneficial *Eubacterium* spp. (e.g., *E. hallii*, *E. coprostanoligenes*, *E. ventriosum*, *E. rectale*, and *E. eligens*) protect against atherosclerosis; increase lipid metabolism^7,37^; produce SCFAs, particularly butyrate, acetate, and propionate^36^; and reduce the contents of proinflammatory cytokines^37^. *E. hallii* is a known butyrate producer^38^. *E. coprostanoligenes* converts cholesterol to fecal coprostanol, mitigating atherosclerosis^39^, so does *E. eligens*^40^. Therefore, the hypocholesterolemic activity of *Eubacterium* can be applied against CVD. In contrast, previous studies have reported that certain *Eubacterium* spp. exert pathogenic effects that influence cholesterol and inflammation levels, as well as other metabolic pathways e.g., obesity^41,42^, and metabolism-associated fatty liver disease^43^.

We observed a markedly increased abundance of an unidentified *Eubacterium* spp. in the CHD group compared to the N and H groups (Fig. 3b; Table 2). We were unable to identify this *Eubacterium* group to the species level. This is a common limitation of *16S rRNA* gene sequencing because the level of heterogeneity may be insufficient for species or subspecies resolution. Furthermore, *16S rRNA* gene sequencing often fails to identify microbial taxa that have low levels of abundance^3^. To improve our findings, we have explored the ASV-level sequences underlying the *Eubacterium* genus-level signal and analyzed closet reference BLASTn matching. The taxonomic assignment revealed that the *Eubacterium* unidentified spp. possess sequence similarity to three distinct ASVs (EUC_1–EUC_3) assigned to *Eubacterium*. BLAST results. These include EUC_1 matched *Eubacterium callanderi* (100% identity), EUC_3 matched *Eubacterium limosum* (100% identity), while EUC_2 showed the highest similarity to *Eubacterium* sp. G3(2011) (100%) and *E. callanderi* (99.75%), indicating unresolved species-level classification (as shown in Supplementary file S4). The distribution of *Eubacterium*-associated ASV-related sequence across the study groups is shown in Supplementary file S4.

The scatterplots of fecal 2-methylbutyric acid using both raw (Fig. 5c) and log_10_-transformed data were generated (Fig. 5d). The positive association between levels of 2-methylbutyric acid and category groups was significant after log10 transformation (Spearman *r* = 0.5037, *p* = 0.0001). Outlier analysis using the ROUT method (Q = 1%) using GraphPad Prism showed no outliers for 2-methylbutyric acid, which is shown in Supplementary file S5. For sensitivity analysis, the data were log_10_(X + 1) transformed to address the right-skewed distribution and ensure the robustness of the findings. The sensitivity analysis revealed that the 2-methylbutyric acid level was positively associated with CHD.

We observed that the abundance of *Eubacterium* unidentified spp. with sequence similarity to *E. liposum* was significantly increased in CHD than the N (FDR = 0.0002) and H groups (FDR = 0.0018). Furthermore, the CHD group showed significantly higher levels of 2-methylbutyric acid, a BSCFA, than the N (FDR = 0.0006). Previous studies have reported enrichment of *E. liposum* abundance in patients with Type 2 Diabetes Mellitus^44^, periodontitis^45^, and children with cerebral palsy and epilepsy^46^.

In conclusion, we observed that patients with CHD had a significant abundance of *E. liposum* and 2-methylbutyric acid. Our important limitations include food uptake, body mass index (BMI) and cardiovascular drugs, which interfere with microbiota composition and diversity. Further in-depth studies are needed to draw definitive conclusions.

## Methods

### Study design, study population, and ethical considerations

Before enrollment, all volunteers were screened and selected by a health and dietary questionnaire; all had the five main food groups with appropriate portions, had moderate exercise (3–5 times/week), no smoking, no alcohol, and no addiction. None was vegetarian and had prebiotics or probiotics. All had no underlying diseases, gut inflammation, and had not received any antibiotics 6 months before enrolling in the study.

We performed a cross-sectional study on 55 volunteers, including 24 healthy controls who did not harbor any infections or underlying diseases or CVD risk factors, and 31 patients with hyperlipidemia or CHD who were diagnosed, classified, and treated by a specialist (KP) at Phra Mongkut Klao Hospital, Bangkok, Thailand. The patients were diagnosed based on their clinical manifestations according to the 2013 American College of Cardiology/American Heart Association criteria^47^. The hyperlipidemia (H) group (*n* = 17) included patients with high cholesterol levels (TC, LDL, and high-density lipoprotein [HDL]), but no evidence of vital organ dysfunction. The CHD group (*n* = 14) included patients diagnosed with CHD, who were started on lipid- and cholesterol-lowering and antiplatelet medications immediately after diagnosis. None of the hyperlipidemia patients or controls had received any cholesterol or blood pressure-lowering medication.

### Sample collection

Whole blood was sampled from healthy controls and from patients before receiving hyperlipidemia treatment or coronary artery bypass graft surgery. The serum from the clotted blood was used for lipid profile tests (TC, triglycerides [TGs], HDL cholesterol [HDL-c], and LDL cholesterol [LDL-c]). Feces samples were collected in stool specimen containers and stored at − 70 °C for analyzing gut microbiota composition and SCFA/BSCFA levels.

### Lipid tests

Lipid markers, including TC, TGs, LDL-c, and HDL-c, were examined enzymatically, using commercial kits (Randox Laboratories Ltd., Crumlin, UK) and an Architect ci16200 biochemistry analyzer (Abbott Laboratories, Abbott Park, IL, USA).

### Fecal microbiota analysis

#### DNA extraction from feces

We used the QIAamp^®^ PowerFecal^®^ Pro DNA Kits (Qiagen, Hilden, Germany) to extract the total DNA from 250 mg of each fecal sample, according to the manufacturer’s instructions. Briefly, each sample was placed into a PowerBead Pro tube and lysed with a TissueLyser LT instrument at 25 Hz for 10 min to achieve homogenization. Then, non-DNA organic and inorganic materials, including polysaccharides, cell debris, and proteins, were removed employing inhibitor removal technology (IRT reagent). The purified DNA was separated from the lysate on an MB spin column, eluted with sterile water, and stored at − 20 °C.

#### 16S rRNA-based metagenomics

The purified DNA was used to prepare a 16 S metagenomic sequencing library. Briefly, the *16S rRNA* gene was amplified using the 2× SparQ HiFi PCR Master Mix (QuantaBio, Beverly, MA, USA) and the primer pair—341 F and 805R—to target the V3–V4 variable region. The PCR cycling steps were initial denaturation at 98 °C for 2 min; 28 cycles of 98 °C for 20 s, 60 °C for 30 s, and 72 °C for 30 s; and final extension at 72 °C for 1 min. Subsequently, the amplicons were purified using SparQ PureMag Beads (QuantaBio) and indexed using 5 µl of each Nextera XT primer (Illumina Inc., San Diego, CA, USA) in a 50 µl PCR reaction, *via* 10 PCR cycles as described above. The products were purified, pooled, and diluted to a loading concentration of 4 pM. Cluster generation and 250-bp paired-end read sequencing were performed on a MiSeq system (Illumina Inc.) at the Omics Sciences and Bioinformatics Center, Chulalongkorn University, Bangkok, Thailand. The sequences of the primers used are shown in Supplementary file 6.

#### Bioinformatics

Bioinformatic analysis of the fecal microbiome, including data processing, operational taxonomic unit (OTU) and diversity analysis, and identifying and differentiating microbiota, was performed using QIIME 2 v2022.11 software (https://qiime2.org)^48^. Briefly, raw sequence data were demultiplexed and quality filtered employing the q2-demux plugin, followed by denoising with DADA2^49^ (via q2‐dada2). A phylogeny was constructed using the SEPP q2-plugin by inserting short sequences into the sepp-refs-gg-13-8.qza reference phylogenetic tree^50^. Alpha‐diversity metrics: observed OTUs and Faith’s phylogenetic diversity^51^; beta-diversity metrics (unweighted Unifrac)^52^; and principal coordinate analyses were ascertained utilizing q2‐diversity after rarifying the samples (subsampled without replacement) to 38,828 sequences per sample. Taxonomy was assigned to the amplification sequence variants employing the q2‐feature‐classifier^53^ classify sk-learn naïve Bayes taxonomy classifier against the Silva 138 99% OTUs reference sequences. The microbiome composition was assessed to identify the differential taxa between groups.

### Preparation of fecal samples and standard solutions for SCFA and BSCFA determination

We extracted SCFAs/BSCFAs from the feces samples. Briefly, we added 20 µl of methanol (MeOH; cat. no. 67-56-1; Sigma-Aldrich, Burlington, MA, USA), 20 µl of 0.5 M NaOH (cat. no. 1310-73-2; Merck, Darmstadt, Germany), and 60 mg of 0.5 mm glass beads (cat. no. 13116-50; Qiagen PowerBead, Qiagen) to 40 mg of feces in a microcentrifuge tube and homogenized the mixture with a TissueLyser LT instrument (Qiagen) at 25 Hz for 5 min. Next, we added 400 µl of 80% MeOH, mixed for 30 s, and incubated at − 20 °C for 30 min. The samples were ultrasonicated on an ice bath for 10 min and centrifuged at 21,500 × *g* for 15 min at 4 °C. Then, 700 µl of the supernatant was transferred into a fresh microcentrifuge tube and dried in a vacuum concentrator at 37 °C for 4 h. The derivatized samples were added with 40 µl of 20 mg/ml methoxyamine hydrochloride (cat. no. 226904; Sigma-Aldrich) and placed in a heat block at 60 °C for 90 min. Then, 60 µl of MTBSTFA (cat. no. 394882; Sigma-Aldrich) and pyridine (cat. no. 360570; Sigma-Aldrich) were added, and the samples were incubated at 60 °C for 30 min, mixed for 30 s, and centrifuged at 22,000 × *g* for 10 min at 4 °C. Finally, 70 µl of the supernatant was transferred into a 2-ml glass vial and diluted with 140 µl of pyridine. The samples were subjected to GC-MS analysis. The seven standard solution mixtures used contained 20 µl of hexanoic acid-6, 6, 6-d_3_ (cat. no. 489727; Sigma-Aldrich) as the internal reference, as well as acetic, propionic, isobutyric, butyric, 2-methylbutyric, isovaleric, and valeric acids. Each standard solution was prepared at eight concentrations by diluting the initial with MeOH. The solution was dried in a vacuum concentrator and prepared for GC-MS analysis using the same process as the fecal samples^54^.

### GC-MS analysis and data extraction

The SCFA/BSCFA samples and the standard solution underwent GC-MS analysis (Agilent 7890 A or Agilent 5977B; Agilent Technologies, Santa Clara, CA, USA) using HP-5ms GC columns (part number 19091 S-433; Agilent Technologies). The analytical quality of each SCFA/BSCFA standard solution was assessed before analysis (Fig. 4). For each sample, the injection volume was 1 µl, with a solvent delay time of 5 min. The initial oven temperature was held at 60 °C for 1 min and then gradually increased to 325 °C at a rate of 10 °C/min with a hold of 10 min. Helium was maintained at a constant flow rate of 20 ml/min through the column. The temperatures of the front inlet, transfer line, and electron impact ion source were set to 250 °C, 290 °C, and 230 °C, respectively. Electron energy was set to − 70 eV, and the mass spectral data were collected in full scan mode (m/z 50–600). The GC-MS data were processed with the Agilent Quantitative Analysis software for compound identification, peak selection, and quantification. SCFAs in feces samples were ascertained based on the calibration curve constructed using the corresponding signals of the SCFA/BSCFA standard^54^.

### Statistical analysis

All statistical analyses were performed using GraphPad Prism 8.0 (https://www.graphpad.com). Data were assessed for normality using the Shapiro-Wilk test. The results are presented as the mean ± SEM for normally distributed data and median (range) for nonparametric data. Parametric data between two groups and among three groups were compared employing an unpaired *t*-test and one-way analysis of variance, respectively. Nonparametric data were compared using Mann-Whitney and Kruskal-Wallis tests, respectively. The α-level was set at < 0.05 with a 95% CI. To reduce false-positive results from multiple testing, *p*-values were adjusted using the Benjamini–Hochberg FDR method with Q = 0.05^33^. Some variables with right-skewed distributions were log_10_(X + 1) transformed before analysis. To determine whether group differences were independent of age, age-adjusted multivariable linear regression analyses were performed with the N group used as the reference. Regression coefficients (β), 95% CI, and *p*-values were reported^24,25^. Outliers of 2-methylbutyric acid levels in each group were identified using the ROUT method (Q = 1%). The association between variables was evaluated using Spearman’s rank correlation coefficient. Statistical significance was set at < 0.05 with a 95% CI.

## Supplementary Information

Below is the link to the electronic supplementary material.

Supplementary Material 1

Supplementary Material 2

Supplementary Material 3

Supplementary Material 4

Supplementary Material 5

Supplementary Material 6

## Data Availability

The datasets presented in this study can be found in online repositories. The names of repositories and accession numbers can be found below: NCBI Bioproject, accession no: PRJNA1311264. The used intervention study data are unsuitable for public deposition due to ethical restrictions and the privacy of participant data. Data are available from this study for any interested researcher who meets the criteria for access to confidential data. Yaowapa Maneerat ([yaowapa.man@mahidol.ac.th](mailto: yaowapa.man@mahidol.ac.th)) may be contacted to request study data.
