# Supplementary material for: Gut microbiota and fecal 2-methylbutyric acid in coronary heart disease: a cross-sectional study
Source: Sci Rep. 2026 Apr 22;16:18627. doi: 10.1038/s41598-026-49930-0 (PMC13269937; doi:10.1038/s41598-026-49930-0)
Supplement: Supplementary file 1 — Supplementary Material 1 [file 41598_2026_49930_MOESM1_ESM.pdf]

Supplementary file S1:

Age-adjusted multivariable linear regression analysis of log-transformed *Eubacterium* abundance and 2-methylbutyric acid levels.

Multiple linear regression analyses were performed to evaluate the associations of study groups, including hyperlipidemia (H) and coronary heart disease (CHD), with the outcomes after adjustment for age. The normal (N) group was used as the reference category. Outcomes were log_{10}(X+1)-transformed prior to analysis. The α level was set at <0.05 with a 95% confidence interval (CI). B, regression coefficient; SE, standard error.

Outcome	Variable	β	SE	95% CI	*p*
**$\log_{10}$(Eubacterium+1)**	Age	-0.0021	0.0058	-0.0137 - 0.0094	0.711
	H vs N	-0.0196	0.1562	-0.3333 - 0.2941	0.901
	CHD vs N	**0.6179**	0.2063	0.2038 - 1.032	**0.0042**
**$\log_{10}$(2-methylbutyric acid+1)**	Age	-0.0028	0.0074	-0.0177 - 0.0122	0.711
	H vs N	**0.4231**	0.2039	0.01347 - 0.8327	**0.0432**
	CHD vs N	**1.127**	0.2653	0.5941 - 1.660	**<0.0001**
