# Supplementary material for: Gut microbiota and fecal 2-methylbutyric acid in coronary heart disease: a cross-sectional study
Source: Sci Rep. 2026 Apr 22;16:18627. doi: 10.1038/s41598-026-49930-0 (PMC13269937; doi:10.1038/s41598-026-49930-0)
Supplement: Supplementary file 2 — Supplementary Material 2 [file 41598_2026_49930_MOESM2_ESM.pdf]

Supplementary file S2:

Relative abundance of bacterial taxa identified at phylum, class, genus, and species levels in normal (N),patients with hyperlipidemia (H), and coronary heart disease (CHD) groups.

**Phylum**	**Class**	**Genus**	**Species**	**% Relative abundance**		
				**N**	**H**	**CHD**
Bacteroidota	*Bacteroidia*	*Bacteroides*	*Bacteroidaceae bacterium*	0.0006	0.0001	0.0005
			Bacteroides caccae	0.0003	0.0008	0.0038
			Bacteroides cellulosilyticus	0.0017	0.0004	0.0008
			Bacteroides clarus	0.0023	0.0008	0.0023
			Bacteroides coprocola	0.0105	0.0065	0.0000
			Bacteroides coprophilus	0.0022	0.0102	0.0170
			Bacteroides cutis	0.0002	0.0003	0.0001
			Bacteroides dorei	0.0096	0.0014	0.0114
			Bacteroides eggerthii	0.0045	0.0010	0.0031
			Bacteroides finegoldii	0.0023	0.0019	0.0037
			Bacteroides fluxus	0.0016	0.0000	0.0000
			Bacteroides fragilis	0.0015	0.0017	0.0035
			Bacteroides intestinalis	0.0004	0.0001	0.0004
			Bacteroides massiliensis	0.0075	0.0070	0.0216
			Bacteroides nordii	0.0003	0.0003	0.0009
			Bacteroides plebeius	0.0232	0.0232	0.0859
			Bacteroides salyersiae	0.0010	0.0005	0.0009
			Bacteroides stercorirosoris	0.0000	0.0001	0.0008
			Bacteroides stercoris	0.0176	0.0341	0.0656
			Bacteroides uniformis	0.0001	0.0006	0.0003
			Bacteroides vulgatus	0.0149	0.0121	0.0422
			uncultured bacterium	0.0000	0.0026	0.0000
			no found data	0.0440	0.0372	0.0945
		Parabacteroides	*Parabacteroides faecis*	0.0000	0.0000	0.0000
			Parabacteroides goldsteinii	0.0003	0.0002	0.0006
			Parabacteroides gordonii	0.0000	0.0000	0.0004
			Parabacteroides johnsonii	0.0013	0.0001	0.0008
			Parabacteroides merdae	0.0034	0.0038	0.0049
			Parabacteroides sp.	0.0000	0.0000	0.0001
			no found data	0.0006	0.0005	0.0004
		Prevotella	human gut	0.0011	0.0006	0.0000
			Massiliprevotella massiliensis	0.0004	0.0005	0.0038
			Prevotella copri	0.0263	0.0328	0.0017
			Prevotella stercorea	0.0040	0.0081	0.0134
			Prevotellaceae bacterium	0.0060	0.0000	0.0000
			uncultured Wautersiella	0.0033	0.0004	0.0000
			no found data	0.1581	0.1397	0.0118
Firmicutes	*Bacilli*	*Streptococcus*	*Streptococcus agalactiae*	0.0000	0.0000	0.0000
			Streptococcus anginosus	0.0000	0.0000	0.0028
			Streptococcus mutans	0.0000	0.0000	0.0004

Phylum	Class	Genus	Species	% Relative abundance		
				N	H	CHD
			Streptococcus parasanguinis	0.0009	0.0003	0.0016
			Streptococcus salivarius	0.0138	0.0069	0.0162
			no found data	0.0021	0.0017	0.0081
	Clostridia	*Butyricicoccus*	*Butyricicoccus pullicaecorum*	0.0000	0.0000	0.0001
			Butyricicoccus sp.	0.0000	0.0000	0.0000
			uncultured bacterium	0.0001	0.0002	0.0001
			no found data	0.0041	0.0016	0.0040
		Eubacterium	no found data	0.0001	0.0000	0.0006
		Lachnospira	uncultured bacterium	0.0075	0.0012	0.0010
			no found data	0.0009	0.0006	0.0002
		Romboutsia	no found data	0.0141	0.0068	0.0062
