# Supplementary material for: Gut microbiota and fecal 2-methylbutyric acid in coronary heart disease: a cross-sectional study
Source: Sci Rep. 2026 Apr 22;16:18627. doi: 10.1038/s41598-026-49930-0 (PMC13269937; doi:10.1038/s41598-026-49930-0)
Supplement: Supplementary file 3 — Supplementary Material 3 [file 41598_2026_49930_MOESM3_ESM.pdf]

Supplementary file S3:

Bacterial genera produce important short (SCFA) and branched short-chain fatty acid (BSCFA)

Data are shown as median (minimum-maximum). The comparison of values was determined by the Kruskal–Wallis test or *one-way ANOVA, as appropriate. Multiple comparisons were adjusted using the Benjamini–Hochberg false discovery rate (FDR) procedure (Q = 0.05). Correlations with SCFA/BSCFA were assessed using **Spearman's rank correlation coefficient, except where indicated (***), for which Pearson's correlation coefficient was applied. The α level was set at <0.05 with a 95% CI. (N, normal; H, hyperlipidemia patients; and CHD, patients with coronary heart disease; AA, Acetic acid; PA, Propionic acid; VA, Valeric acid; IBA, Isobutyric acid; 2-MBA, 2-Methylbutyric acid, IVA, Isovaleric acid)

Phylum/Genus	N<br>(n=24)	H<br>(n=17)	CHD<br>(n=14)	*p*	FDR (q)	Sig. pairs	Correlation** with SCFA/BSCFA						
							AA	PA***	BA	VA	IBA	2-MBA	IVA***
**p_*Actinobacteriota***													
Bifidobacterium	337<br>(10-2571)	178<br>(7-2553)	251<br>(42-798)	ns	ns	-	-0.08043<br>(0.5632)						
Collinsella	540.5<br>(0-1376)	718<br>(6-1548)	350.5<br>(0-4984)	ns*	ns	-			-0.0681<br>(0.6245)				
**p_*Bacteroidota***													
Alistipes	241<br>(0-2360)	410<br>(11-1938)	754.5<br>(49-2039)	ns	ns	-		**-0.2698<br>(0.0485)**					
Bacteroides	4710<br>(287-16076)	5025<br>(435-17076)	15358<br>(4496-23456)	**0.0007***	**<0.0001**	N vs CHD<br>H vs CHD	-0.04471<br>(0.7482)	**-0.2989<br>(0.0281)**			-0.04982<br>(0.7205)		-0.0051<br>(0.9708)
Butyricimonas	82.5<br>(0-1139)	29<br>(0-252)	154.5<br>(0-536)	ns	ns	-			-0.0848<br>(0.5423)				
Odoribacter	56<br>(0-563)	120<br>(0-791)	116<br>(0-2203)	ns	ns	**-**	**0.2451<br>(0.074)**	-0.0641<br>(0.6451)	0.0615<br>(0.6585)				
Parabacteroides	94<br>(0-1522)	107<br>(11-811)	212<br>(9-958)	**0.0492**	ns	-	**0.3566<br>(0.0081)**			**0.2886<br>(0.0343)**	**0.356<br>(0.0082)**		**0.3261<br>(0.0161)**
Paraprevotella	34.5<br>(0-1950)	104<br>(0-2794)	0<br>(0-2250)	ns	ns	-			0.1411<br>(0.3087)				
Porphyromonas	0<br>(0-126)	0	0	ns	ns	-		-0.0183<br>(0.8957)					
Prevotella	3272<br>(0-30126)	3502<br>(0-26892)	0<br>(0-6728)	**0.0016**	**0.0024**	N vs CHD<br>H vs CHD		**0.5375<br>(<0.0001)**					

Phylum/Genus	N<br>(n=24)	H<br>(n=17)	CHD<br>(n=14)	*p*	FDR (q)	Sig. pairs	Correlation** with SCFA/BSCFA						
							**AA**	**PA*****	**BA**	**VA**	**IBA**	**2-MBA**	**IVA*****
**p_*Desulfobacterota***													
Desulfovibrio	0<br>(0-349)	18<br>(0-620)	4.5<br>(0-355)	ns	ns	-	0.1835<br>(0.184)						
**p_*Firmicutes***													
[Clostridium]	0<br>(0-9)	0<br>(0-41)	1.5<br>(0-38)	ns	ns	-					0.0845<br>(0.5435)	0.03951<br>(0.7767)	0.0583<br>(0.6752)
Enterococcus	17<br>(0-264)	15<br>(0-15)	0<br>(0-190)	ns	ns	-	0.1377<br>(0.3206)						**0.4718<br>(0.0003)**
Holdemanella	6<br>(0-1239)	52<br>(0-609)	0<br>(0-356)	ns	ns	-			0.0566<br>(0.6846)				
Lactobacillus	0<br>(0-528)	9<br>(0-1428)	39<br>(0-6787)	ns	ns	-	0.224<br>(0.1034)			**0.3421<br>(0.0113)**	0.2196<br>(0.1105)	0.1787<br>(0.196)	**0.446<br>(0.0007)**
Streptococcus	103.5<br>(3-10111)	78<br>(13-4205)	796.5<br>(100-3674)	**0.0012**	**0.0032<br>0.0018**	N vs CHD<br>H vs CHD	0.1656<br>(0.2315)						**0.2967<br>(0.0294)**
Eubacterium hallii group	279<br>(0-929)	291<br>(0-1675)	151<br>(0-1185)	ns	ns	-	0.0047<br>(0.9729)	-0.0683<br>(0.6236)	-0.0859<br>(0.5367)	0.0491<br>(0.7246)			
Ruminococcus	60.5<br>(0-1821)	237<br>90-4017)	44.5<br>(0-1226)	ns	ns	-	-0.0966<br>(0.487)		0.1904<br>(0.168)				
Agathobacter	198.5<br>(0-3074)	310<br>(0-2924)	160.5<br>(0-2460)	ns	ns	-	-0.184<br>(0.183)						**-0.3293<br>(0.015)**
Anaerostipes	233<br>(0-40180	182<br>(0-958)	148<br>(0-1530)	ns	ns	-	**-0.3638<br>(0.0069)**		0.0422<br>(0.762)		**-0.3601<br>(0.0075)**		**-0.3126<br>(0.0214)**
Blautia	1921<br>(158-5458)	1950<br>(417-11211)	1606<br>(495-6293)	ns	ns	-	-0.1912<br>(0.166)	-0.1918<br>(0.1648)	**-0.3621<br>(0.0071)**				
Butyricicoccus	132<br>(19-796)	64<br>(0-209)	84<br>(0-1235)	**0.0116**	**0.0096**	N vs H			-0.2103<br>(0.127)				
Butyrivibrio	0<br>(0-60)	0	0	ns	ns	-	-0.119<br>(0.3915)		0.119<br>(0.3915)				
Clostridia	0<br>(0-227)	4<br>(0-61)	0<br>(0-653)	ns	ns	-	0.0951<br>(0.4942)		0.0521<br>(0.7085)				
Clostridium sensu stricto 1	34<br>(0-11190)	37<br>(0-797)	22<br>(0-1131)	ns	ns	-					-0.1522<br>(0.272)	-0.0263<br>(0.8502)	-0.2065<br>(0.1341)
Coprococcus	190<br>(0-997)	200<br>(0-744)	1<br>(0-483)	ns	ns	-		-0.0286<br>(0.8374)	-0.0637<br>(0.6473)			**-0.3318<br>(0.0142)**	

Phylum/Genus	N<br>(n=24)	H<br>(n=17)	CHD<br>(n=14)	*p*	FDR (q)	Sig. pairs	Correlation** with SCFA/BSCFA						
							AA	PA***	BA	VA	IBA	2-MBA	IVA***
Dorea	446<br>(0-927)	410<br>(114-1645)	286<br>(0-1665)	ns	ns	-	0.0177<br>(0.8992)	-0.1454<br>(0.2942)	-0.1788<br>(0.1958)			**-0.2703<br>(0.048)**	
Eubacterium	0<br>(0-101)	0<br>(0-10)	2.5<br>(0-224)	**0.0002**	**0.0003<br>0.0009**	N vs CHD<br>H vs CHD	0.1995<br>(0.1481)		-0.1336<br>(0.3353)			**0.3947<br>(0.0031)**	**0.3456<br>(0.0105)**
Faecalibacterium	2486<br>(0-5629)	1732<br>(9-12134)	932.5<br>(0-3797)	ns	ns	-			0.157<br>(0.2569)			**-0.3676<br>(0.0062)**	
Fusicatenibacter	379.5<br>(0-1808)	385<br>(91-3157)	572<br>(0-2012)	ns	ns	-			0.02159<br>(0.8769)				
Intestinimonas	0<br>(0-43)	0<br>(0-14)	0<br>(0-39)	ns	ns	-		-0.1656<br>(0.2315)					
Lachnoclostridium	105<br>(13-1238)	87<br>(9-345)	83<br>(11-397)	ns	ns	-		-0.1861<br>(0.1778)	**-0.2856<br>(0.0363)**	**-0.318<br>(0.0191)**	**-0.3044<br>(0.0252)**		
Lachnospira	66<br>(0-4102)	71<br>(0-179)	25.5<br>(0-382)	**0.0238**	**0.0192**	N vs CHD		0.136<br>(0.3269)	**0.3406<br>(0.0117)**			**-0.2896<br>(0.0336)**	
Lachnospiraceae	304<br>(0-1762)	245<br>(0-1059)	121.5<br>(0-3591)	ns	ns	-		0.0972<br>(0.4844)	0.2392<br>(0.0815)				
Negativibacillus	8.5<br>(0-453)	0<br>(0-231)	74<br>(0-327)	ns	ns	-	**0.3938<br>(0.0032)**	-0.0598<br>(0.6677)	-0.1479<br>(0.2859)	**0.302<br>(0.0265)**	**0.3972<br>(0.0029)**		**0.3698<br>(0.0059)**
Peptococcus	0	0<br>(0-170)	0<br>(0-21)	ns	ns	-	0.1475<br>(0.2872)		-0.09628<br>(0.4886)				
Romboutsia	85.5<br>(0-6270)	172<br>(0-988)	17<br>(0-2775)	**0.0224**	**0.0228**	N vs CHD	-0.0685<br>(0.6226)						
Roseburia	542.5<br>(0-4695)	351<br>(13-1994)	455<br>(0-1698)	ns	ns	-		0.1104<br>(0.4269)	**0.4574<br>(0.0005)**				**-0.29<br>(0.0334)**
Ruminococcus	60.5<br>(0-1821)	237<br>(0-4017)	44.5<br>(0-1226)	ns	ns	-			0.1904<br>(0.168)				
Subdoligranulum	160.5<br>(0-1237)	248<br>(0-2664)	68<br>(0-1867)	ns	ns	-			0.0673<br>(0.6287)	**0.2736<br>(0.0453)**			**0.2916<br>(0.0324)**
Acidaminococcus	0<br>(0-1115)	0<br>(0-1226)	0<br>(0-2455)	ns	ns	-			-0.146<br>(0.2923)	**0.2777<br>(0.042)**			**0.5025<br>(0.0001)**
Dialister	0<br>(0-1427)	0<br>(0-1529)	0<br>(0-633)	ns	ns	-		-0.00616<br>(0.9647)					
Megamonas	9.5<br>(0-27551)	0<br>(0-28838)	0<br>(0-4008)	ns	ns	-	0.03876<br>(0.7808)	-0.1222<br>(0.3787)	-0.1974<br>(0.1525)				

Phylum/Genus	N (n=24)	H (n=17)	CHD (n=14)	*p*	FDR (q)	Sig. pairs	Correlation** with SCFA/BSCFA						
							**AA**	**PA***	**BA**	**VA**	**IBA**	**2-MBA**	**IVA***
Megasphaera	0 (0-2704)	0 (0-1847)	0 (0-1223)	ns	ns	-		-0.1414 (0.3079)					
Phascolarctobacterium	514 (0-2991)	718 (0-2848)	769 (0-3558)	ns	ns	-		-0.1907 (0.1673)					
Veillonella	17 (0-963)	5 (0-1218)	18.5 (0-2741)	ns	ns	-		-0.0163 (0.907)	0.2194 (0.111)				
**p_*Fusobacteriota***													
Fusobacterium	0 (0-2737)	0 (0-196)	23 (0-2344)	ns	ns	-	-0.2163 (0.1162)		-0.07962 (0.5671)				
Enterobacter	6.5 (0-2361)	14 (0-308)	1.5 (0-102)	ns	ns	-	-0.2097 (0.1281)						
Escherichia-Shigella	76 (0-2034)	138 (7-921)	363.5 (23-6111)	ns	ns	-	0.1641 (0.2358)						**0.3174 (0.0194)**
Sutterella	247.5 (0-1471)	86 (0-1286)	92 (0-1127)	ns	ns	-	0.2265 (0.0996)	0.0356 (0.7986)	0.2107 (0.1261)				
**p_*Verrucomicrobiota***													
Akkermansia	0 (0-3532)	0 (0-902)	0 (0-163)	ns	ns	-	0.0122 (0.9305)	-0.1005 (0.4696)	**-0.3804 (0.0046)**				
