# Supplementary material for: Gut microbiota and fecal 2-methylbutyric acid in coronary heart disease: a cross-sectional study
Source: Sci Rep. 2026 Apr 22;16:18627. doi: 10.1038/s41598-026-49930-0 (PMC13269937; doi:10.1038/s41598-026-49930-0)

Supplementary file S4:

ASV-level distribution of *Eubacterium*-associated sequences across study groups.

Distribution of three *Eubacterium*-associated ASVs (ASV_EUC1–ASV_EUC3) across the normal (N), patients with hyperlipidemia (H), and coronary heart disease (CHD) groups. (a) Relative abundance of ASV_EUC1–ASV_EUC3 in each group. Data are shown as raw relative abundance and summarized as median (min–max). Group differences were tested using the Kruskal–Wallis test on log_{10}(x+1)-transformed data, and q-values were calculated using the Benjamini–Hochberg false discovery rate (FDR). (b) Comparison of ASV abundances among groups shown by FDR-adjusted q-values.

a

**ASV ID**	**Closest BLAST hit**	**N** (n=24)	**H** (n=17)	**CHD** (n=14)	***p***	**q(FDR)**		
						**N vs H**	**N vs CHD**	**H vs CHD**
ASV_EUC1	*Eubacterium callanderi*	0 (0-101)	0 (0-0)	0 (0-2)	0.5765	0.6628	0.6628	0.6628
ASV_EUC2	*Eubacterium sp. G3(2011)*	0 (0-0)	0 (0-0)	0 (0-96)	0.2312	>0.9999	0.2132	0.2132
ASV_EUC3	*Eubacterium limosum*	0 (0-2)	0 (0-10)	2 (0-128)	**0.0002**	0.5561	**0.0002**	**0.0018**

b

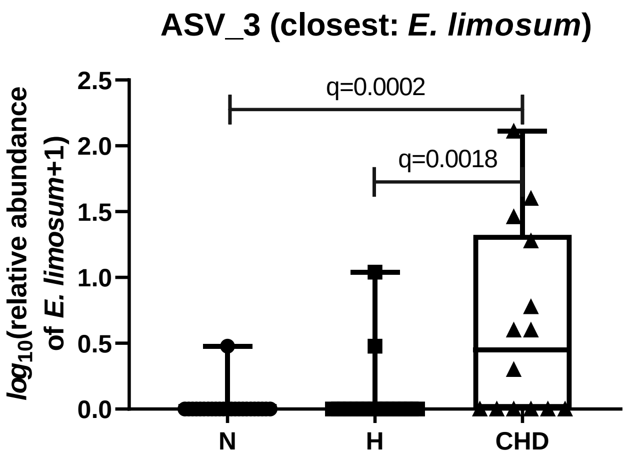

Supplement: Supplementary file 4 — Supplementary Material 4 [file 41598_2026_49930_MOESM4_ESM.pdf]
