# Supplementary material for: Gut microbiota and fecal 2-methylbutyric acid in coronary heart disease: a cross-sectional study
Source: Sci Rep. 2026 Apr 22;16:18627. doi: 10.1038/s41598-026-49930-0 (PMC13269937; doi:10.1038/s41598-026-49930-0)

Supplementary file S5:

Distribution of fecal 2-methylbutyric acid concentrations among normal controls, hyperlipidemia, and coronary heart disease patients groups.

Violin plots show the distribution of log_{10}-transformed 2-methylbutyric acid concentrations (log_{10} [ppm + 1]) in normal controls (N), hyperlipidemia patients (H), and coronary heart disease patients (CHD). Each dot represents an individual sample. The width of the violin indicates the density of observations, and the central symbols represent the median and interquartile range.

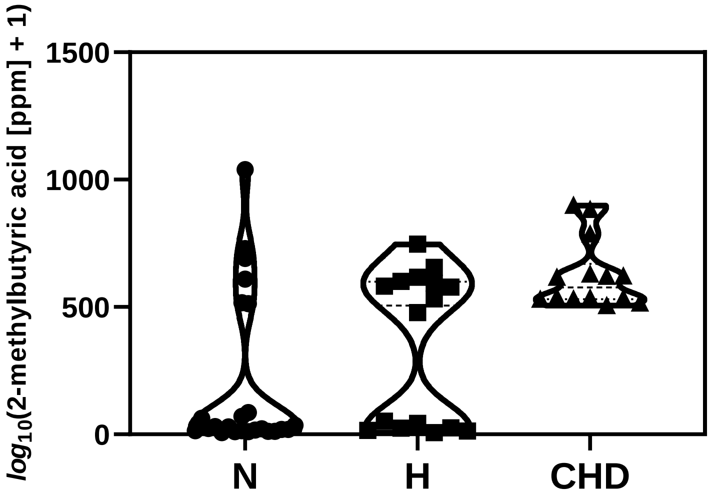

Supplement: Supplementary file 5 — Supplementary Material 5 [file 41598_2026_49930_MOESM5_ESM.pdf]
