# Supplementary material for: Gut microbiota and fecal 2-methylbutyric acid in coronary heart disease: a cross-sectional study
Source: Sci Rep. 2026 Apr 22;16:18627. doi: 10.1038/s41598-026-49930-0 (PMC13269937; doi:10.1038/s41598-026-49930-0)
Supplement: Supplementary file 6 — Supplementary Material 6 [file 41598_2026_49930_MOESM6_ESM.pdf]

Supplementary file S6:

Primer sequence used for 16S V3-V4 amplicon primers.

**Underline sequences are overhang adapter sequences which are trimmed automatically.
Read **bold sequences are 16S V3V4 region.

16S V3-V4 Amplicon primers	
Forward Primer	TCGTCGGCAGCGTCAGATGTGTATAAGAGACAG**CCTACGGGNGGCWGCAG**
Reverse Primer	GTCTCGTGGGCTCGGAGATGTGTATAAGAGACAG**GACTACHVGGGTATCTAATCC**
